# Identification of extracellular miRNA in archived serum samples by next-generation sequencing from RNA extracted using multiple methods

**DOI:** 10.1007/s11033-016-4043-6

**Published:** 2016-08-10

**Authors:** Aarti Gautam, Raina Kumar, George Dimitrov, Allison Hoke, Rasha Hammamieh, Marti Jett

**Affiliations:** 1 US Army Center for Environmental Health Research, 568 Doughten Drive, Fort Detrick, 21702-5010 MD USA; 2 Advanced Biomedical Computing Center, Frederick National Laboratory for Cancer Research/Leidos-Biomedical Inc., Frederick, MD 21702 USA; 3 The Geneva Foundation, US Army Center for Environmental Health Research, Fort Detrick, MD 21702 USA

**Keywords:** DoDSR, miRNA, Next-generation sequencing, Serum, Data analysis

## Abstract

miRNAs act as important regulators of gene expression by promoting mRNA degradation or by attenuating protein translation. Since miRNAs are stably expressed in bodily fluids, there is growing interest in profiling these miRNAs, as it is minimally invasive and cost-effective as a diagnostic matrix. A technical hurdle in studying miRNA dynamics is the ability to reliably extract miRNA as small sample volumes and low RNA abundance create challenges for extraction and downstream applications. The purpose of this study was to develop a pipeline for the recovery of miRNA using small volumes of archived serum samples. The RNA was extracted employing several widely utilized RNA isolation kits/methods with and without addition of a carrier. The small RNA library preparation was carried out using Illumina TruSeq small RNA kit and sequencing was carried out using Illumina platform. A fraction of five microliters of total RNA was used for library preparation as quantification is below the detection limit. We were able to profile miRNA levels in serum from all the methods tested. We found out that addition of nucleic acid based carrier molecules had higher numbers of processed reads but it did not enhance the mapping of any miRBase annotated sequences. However, some of the extraction procedures offer certain advantages: RNA extracted by TRIzol seemed to align to the miRBase best; extractions using TRIzol with carrier yielded higher miRNA-to-small RNA ratios. Nuclease free glycogen can be carrier of choice for miRNA sequencing. Our findings illustrate that miRNA extraction and quantification is influenced by the choice of methodologies. Addition of nucleic acid- based carrier molecules during extraction procedure is not a good choice when assaying miRNA using sequencing. The careful selection of an extraction method permits the archived serum samples to become valuable resources for high-throughput applications.

**Electronic supplementary material:**

The online version of this article (doi:10.1007/s11033-016-4043-6) contains supplementary material, which is available to authorized users.

## Introduction

miRNA is a functionally important class of small, non–protein coding RNA, 19–24 nucleotides in length, that are post-transcriptional regulators of gene expression [1]. miRNAs act as important regulators of gene expression by promoting mRNA degradation or by attenuating protein translation [2]. It has been suggested that miRNA can potentially regulate every aspect of cellular activity such as cell development, differentiation, proliferation, cell death, stress reaction, fat metabolism, insulin secretion and carcinogenesis [3–5]. A single miRNA can regulate hundreds of mRNAs by recognizing complementary sequences in the 3′UTRs of their target mRNA, and multiple miRNAs can regulate an individual mRNA [6]. Current estimates indicate that more than one-third of the cellular transcriptome is regulated by miRNAs [7]. miRNAs have been found in tissues, serum/plasma, and other body fluids in a stable form that is protected from endogenous RNase activity. miRNA expression in biofluids, such as serum, plasma, urine or cerebrospinal fluid, is a rapidly expanding area of research [8]. miRNAs in circulation are thought to contribute to the normal functioning of circulatory and immune systems [9, 10] and have been proposed as candidate biomarkers of health/disease status [11, 12]. Results of another study by Keller and coworkers [13] strengthen the potential of blood-borne miRNAs as biomarkers. The use of serum or plasma reduces variables and possibly allows the detection of a more concentrated disease-related miRNome by decreasing the background expression of miRNAs from blood cells.

The measurement and exact quantification of miRNAs from serum are hampered by low yields of RNA due to its low concentration (0.1–1 ng/ml) [14]. However, miRNA is considered stable even after subjected to severe conditions such as freeze–thaw cycles and storage conditions, as well as changes in the pH levels [15, 16]. In contrast, synthetic, unbound, free-circulating miRNA degrades rapidly [17]. The profiles of miRNA are preserved up to 10 years in archived serum [18], and it was recently published that the miRNA and DNA may be frozen up to 40 years without significant degradation [19, 20]. A technical hurdle in studying the dynamics of gene expression is the ability to reliably extract miRNA from these compromised biological samples. However, a number of commercial extraction kits have recently become available that seek to optimize the extraction of small RNAs, either in conjunction with full length total RNA or as a fraction enriched for small RNAs. Historically, genome-wide analysis of miRNA expression has been performed through microarrays [21, 22], but the effort is now largely based on sequencing [23–26]. Next-Generation Sequencing (NGS) technology has quickly emerged as a preferred method for studying miRNA expression. Sequencing provides the opportunity to examine all miRNAs simultaneously and has the potential to identify novel miRNAs. Thus, isolation of miRNAs must be optimized for sequencing analysis.

In this study, we evaluated different extraction procedures, with and without addition of a carrier, on serum “test samples” that were obtained from the Department of Defense Serum Repository (DoDSR). These specimens are linked with the Defense Medical Surveillance System (DMSS) data and are augmented by epidemiological studies for military and operational relevance, and upon additional approval, the samples can be applied for research purposes. The rich details related to military service that have been collected provide health professionals with unique opportunities to utilize these archived samples to find solutions for both demographical groups and individuals. It is important to optimize the different aspects of sample management, such as sample storage conditions and extraction procedures, and to demonstrate that the miRNA obtained is of sufficient quality for NGS.

## Materials and methods

### Serum samples

**Fig. 1:**
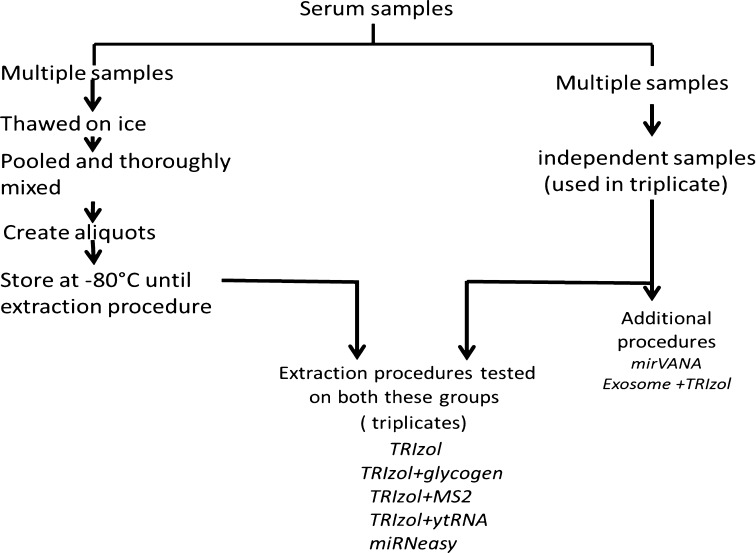
Experimental set-up: Multiple pooled serum samples, as well as an independent set of unpooled serum samples, were used for RNA extractions using multiple methods

The serum samples were obtained from the DoDSR where they were stored at −30 °C. These samples were called “test samples” and were used to conduct a pilot study with the purpose of determining suitable methods for these compromised samples before setting up a larger study. In the current study, a subset of samples was thawed on ice, pooled, and divided into 350 μL aliquots, frozen and stored at −80 °C to be used in extractions as “pooled” samples (Fig. 1) and we used a subset of independent, archived serum samples as received from the DoDSR, calling this set “unpooled samples.” For each extraction method, individual 350 µL sample aliquots were thawed, followed by addition of initial denaturant from the respective kits. We used triplicates samples for each extraction therefore a total of 36 samples were generated from the study. All procedures used 350 μL of serum for miRNA extractions except the Qiagen miRNeasy Mini Kit, where 200 μL of sample was used per extraction.

### RNA extraction

Different miRNA extraction methods were evaluated with either unpooled or pooled serum samples used in triplicate per extraction method. The extraction methods TRIzol Reagent (Invitrogen, Life Technologies, Grand Island, NY, USA) and TRIzol LS Reagent (Invitrogen) were tested with or without a different carrier, such as glycogen (Invitrogen), bacteriophage MS2 (Roche, Basel, Switzerland) and yeast tRNA (Invitrogen). Extractions were also performed using the miRNeasy Mini Kit (Qiagen, Valencia, CA, USA). Other extraction methodologies that were tested only on the unpooled samples were the mirVANA miRNA Isolation Kit (Ambion, Life Technologies, Thermo Fisher Scientific, Waltham, MA, USA), the ExoQuick Exosome precipitation (System Biosciences, Inc., Mountain View, CA, USA) extraction in conjunction with the TRIzol procedure, and the ExoQuick Exosome precipitation in combination with mirVANA miRNA Isolation Kit. The detailed study plan is shown in Fig. 1. The description of each extraction method is explained below.

### TRIzol

TRIzol extractions were conducted according to the manufacturer’s protocol (Invitrogen). Briefly, 700 μL of TRIzol was added to 350 μL of serum followed by incubation at room temperature for 5 min. Chloroform (Sigma-Aldrich, St. Louis, MO, USA) was added, and the samples were vortexed and incubated for 5 min at room temperature followed by centrifugation at 12,000×*g* for 15 min at 4 °C. The aqueous phase containing RNA was transferred to a new tube and isopropanol (Fisher-Scientific, Thermo Fisher Scientific, Waltham, MA, USA) was added. The samples were incubated at room temperature for 10 min followed by centrifugation at 12,000x*g* for 15 min at 4 °C. The pellet was washed with 75 % Ethanol (Sigma-Aldrich), air-dried and resuspended in 20 μL nuclease-free water (Ambion). For addition of carriers, 2 μL of glycogen (5 μg/μL), 1 μL of bacteriophage MS2 RNA (500 ng/μL) or 1 μL of yeast tRNA (500 ng/μL) were added during the isopropanol precipitation of the extraction procedure. TRIzol LS was used instead of TRIzol in the procedure using the glycogen carrier.

### mirVANA kit

The samples were processed using the mirVANA miRNA Isolation Kit following the manufacturer’s protocol.

### ExoQuick exosome precipitation followed by TRIzol extraction

The samples were processed using the ExoQuick Exosome precipitation method by adding 90 μL of ExoQuick solution (System Biosciences, Inc.) and incubating overnight at 4 °C. The samples were centrifuged at 1500x*g* for 30 min at room temperature and the supernatant was aspirated, followed by centrifugation of the pellet at 1500x*g* for 5 min at room temperature to remove all traces of residual Exoquick solution. The pellet was dissolved in 350 μL of TRIzol reagent and the TRIzol extraction procedure was followed.

### miRNeasy kit

The miRNeasy Mini Kit was used to extract miRNA and total RNA following the manufacturer’s protocols.

### miRNA quality control

The quality of the RNA was evaluated by the Bioanalyzer 2100 instrument (Agilent, Santa Clara, CA, USA) using the small RNA chip (Agilent) according to the manufacturer’s protocol.

### Small RNA sequencing

Small RNA libraries were prepared using the TruSeq Small RNA Sample Preparation kit (Illumina, San Diego, CA, USA) following the manufacturer’s instructions. A fraction of five microliter of total RNA was used for library preparation as quantification is below the detection limit. Five microliters of total RNA was ligated with 3′ and 5′ adaptors followed by reverse transcription with SuperScript II Reverse Transcriptase (Invitrogen) and unique indexes were introduced during PCR amplification for 15 cycles. A multiplexed pool consisting of equimolar amounts of small RNA-derived libraries was prepared. The 140–150 nt region containing the microRNA fraction of the library was excised from a 6 % polyacrylamide gel after electrophoresis, ethanol-precipitated and quantified. The samples were subsequently sequenced on the Illumina HiScan SQ instrument using 50 + 7 (index) sequencing cycles.

### Sequencing data analysis

**Fig. 2:**
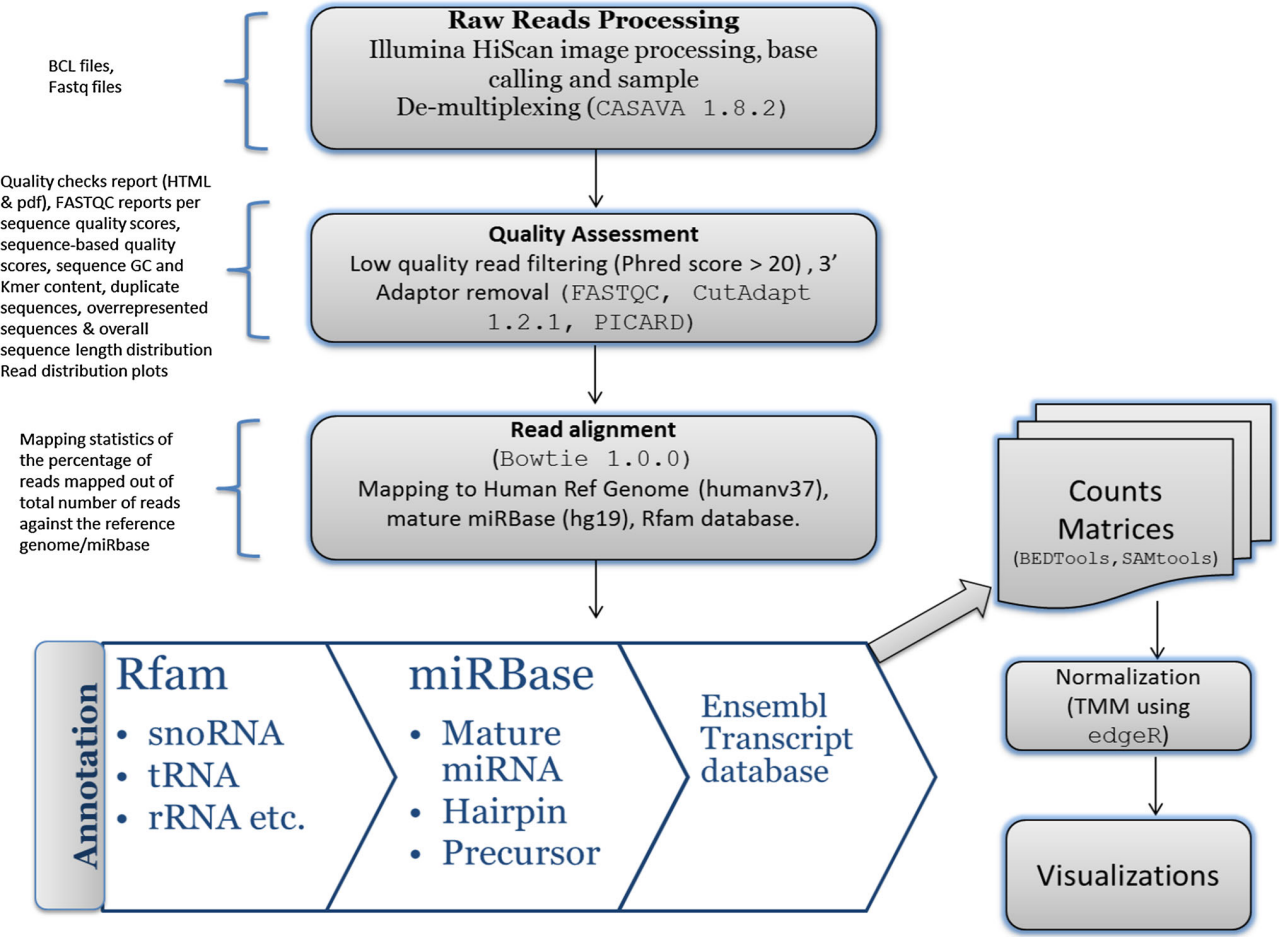
Pipeline for data analysis: Raw reads are preprocessed for quality assessment, quality filtering and adaptor trimming of raw reads, followed by sequence read alignment against reference genome assembly and miRNA quantification, and lastly, expression and functional analysis. The quality control step generates quality check reports in HTML/PDF format. The reports highlight the per sequence quality scores, sequence base quality scores, sequence GC and Kmer content, duplicate sequences, overrepresented sequences and overall sequence length distribution. The alignment step generates alignment files and HTML files with mapping statistics for the percentage of reads mapped out of total number of reads against reference genome/miRBase. This also generates the count matrix of total read count for each annotated miRNA/gene for each sample in text file format

Our data analysis pipeline (Fig. 2) performs preprocessing of reads for quality assessment, quality filtering, and adaptor trimming of raw reads, then implements sequence read alignments against reference genome assembly, miRNA quantification, and lastly, executes expression and functional analyses.

The standard open source tool CASAVA 1.8.2 (Illumina, Inc.) was used to preprocess raw base calls and de-multiplex samples. Reads were processed further for quality assessment, quality filtering, and adaptor trimming, and quality filtered again using a Phred quality score threshold of 20. The ligated adaptor sequences were trimmed, leaving a minimum overlap of five bases while allowing an error rate of 0.1 using CutAdapt 1.2.1. (Martin 2011). Additional filtering was also applied to retain sequences with a minimum length of 15 base pairs and to discard sequences of fewer than 15 base pairs in length. The average read length distribution across protocols per purification method was further statistically assessed using Pearson Chi Square test using read length as bins for each protocol.

Using the short read aligner Bowtie 1.0.0 [27], filtered sequence reads were aligned to the Human reference genome assembly humanv37 from Ensembl [28], and the best hits to the genome were identified using BEDtools 2.17. Small RNA species were annotated by mapping with the reference gene feature annotation table from the Ensembl database. Quantification/enrichment reads for miRNAs were first aligned to the Rfam RNA families database to filter other small RNA species, and then reads were mapped for small RNA annotation against reference mature/precursor hg19_mature RNA sequences from the miRBase (miRbase 19) database [29–32]. The miRNA read count matrix for each sample was generated using open source tools Picard 1.84 (http://broadinstitute.github.io/picard), SAMtools 0.1.19 [33], BEDtools 2.17 [34] and shell/Perl/R scripts [35]. The miRNA count was normalized using TMM normalization method using R Biocondutor package edgeR. The Excel CORREL function that calculates the Pearson Product-Moment was used to calculate the correlation coefficient to determine the relationship between replicates isolated using different extraction methods. The sequencing data was submitted to the Gene Expression Omnibus (GEO) and raw reads are available through Sequence Read Archive (SRA) using series #GSE80274 and Platform ID:GPL15456.

## Results and discussion

The purpose of our study was to develop a method of RNA extraction from archived serum samples stored at −30 °C to obtain sufficient yields and acceptable quantities of miRNA for NGS. miRNA has been shown to be present in twelve body fluids tested [36], and higher miRNA concentrations have been observed in serum samples as compared to plasma samples [37]. Serum is known to contain a considerable amount of stable miRNA [15], and in recent years, researchers have proposed that serum miRNAs could possibly serve as a source of novel biomarkers for uncovering various diseases [11, 12]. Considering the collection and storage of serum samples at the DoDSR and the stability of miRNA, we can potentially use these samples for biomarker discovery related to diseases of military relevance.

### RNA extraction procedures

The method used to purify RNA impacts both yield and the spectrum of RNA isolated [38, 39]. miRNA molecules behave physically and chemically different from the larger RNA molecules, and their quantitative recovery requires optimization of existing total RNA isolation procedures. To date, most RNA and miRNA extraction methods use a phenol: chloroform-based extraction technique often facilitated by the addition of guanidinium thiocyanate, and the most commonly used reagent in this technique is TRIzol [40]. This reagent relies on isopropanol precipitation and centrifugation sedimentation. Although TRIzol isolation is a widely employed method, it has been reported that TRIzol favors specific miRNAs and fails to isolate those low in guanine and cytosine [41]. Furthermore, if the RNase-containing denaturing reagents are not removed completely in the final RNA preparation, they can interfere with the efficiency of downstream applications. To remove serum RNases, many commercial kits such as the Qiagen miRNeasy Mini Kit and mirVANA miRNA Isolation Kit use filtering agents that remove the RNA inhibitors during the extraction process. Therefore, it is important to choose an extraction method which minimizes the carry-over of such inhibitors while maximizing the RNA yield. Several previous reports have compared miRNA extraction kits. Notably, Kroh et al., have tested variations of two extraction kits for plasma and serum samples: Ambion’s mirVANA PARIS (with addition of an extra organic extraction step), and the Qiagen miRNeasy Mini Kit (with a protocol modified to use 10 volumes of Qiazol reagent per volume of plasma or serum). They showed that although both protocols have proven to be effective, the Qiagen protocol appears to produce two- to three-fold greater RNA yield [42]. Likewise, Li and colleagues evaluated the performances of the Qiagen miRNeasy kit, the Ambion mirVANA PARIS kit and the Total RNA Purification Kit (Norgen Biotek, Canada) [43]. They concluded that RNAs isolated by the Qiagen or Ambion kits had better quality (in terms of the percentage of miRNAs in the small RNA fraction) than those extracted with the Norgen kit. We tested the TRIzol procedure, the mirVANA miRNA Isolation Kit, and the Qiagen miRNeasy Kit in our study, noting that these kits have been used to assess plasma and cerebrospinal fluid samples [44]. miRNA are also known to exist in exosomes [45], and we utilized the ExoQuick Exosome precipitation method along with TRIzol procedure.

Serum has low amounts of total RNA, and small RNA only constitutes 0.4–0.5 % of this total [46]. To maximize the yield of isolated RNA that includes small RNA, we added known quantities of carrier molecules for precipitation of total RNA. Glycogen, yeast tRNA, and bacteriophage MS2 RNA were added as carriers during the TRIzol extraction procedure.

The use of glycogen in RNA isolation has been reported previously [47] to prepare samples for subsequent real-time PCR analysis of miRNA levels. Glycogen has been reported to be an effective carrier for certain procedures: extraction of 50 µL of plasma using the miRNeasy Mini Kit with the addition of 5 µg of glycogen resulted in the highest recovery of endogenous miRNA [48]. A study of RNA extraction of plasma by different methods found that addition of glycogen seemed to be beneficial for some, but not all, isolation methods tested [44]. Addition of bacteriophage RNA has been observed to improve miRNA quantification [49], but more studies are needed to demonstrate its reliability. Yeast tRNA [50] has been used for isolation of microscale RNA, and the GeneChip RNA control RNA in the analyses confirms that addition of carrier tRNA does not cause detectable distortion in global amplification [51].

Many studies have compared different methods for miRNA extraction [44]. TRIzol-chloroform extraction has been shown to be the most suitable for large-scale experiments [52] and allowed the highest recovery of low molecular weight RNA [53]. Recently, studies of plasma miRNA extraction methodologies comparing TRIzol to column-based procedures revealed that the Qiagen miRNeasy Serum/Plasma Kit rendered more concentrated RNA [50]. This study also reported that the addition of 1 μg/mL yeast tRNA gave better results than 10 μg/mL of yeast tRNA. In all the protocols investigated, we were able to save the DNA phase during extraction. Maximizing DNA and total RNA retrieval from the same specimen is very useful when extracting samples of limited availability.

The samples obtained from the DoDSR cannot be controlled for the initial sample processing and storage conditions; therefore, the purpose of this study was to test for maximum RNA yield using the different isolation methods available.

### RNA quality and yield

**Fig. 3:**
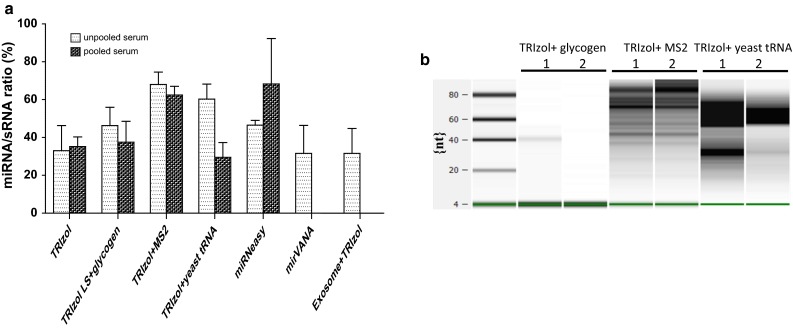
Small RNA analysis performed by using the Agilent 2100 Bioanalyzer and and the sequencing library preparation using Illumina TruSeq Small RNA kit: **a** miRNA/sRNA ratio: Ratio of miRNA molecules expressed as a portion of all small RNA molecules (%) detected with the Agilent 2100 Bioanalyzer. **b** Electronic gel image of small RNA library obtained using Illumina TruSeq Small RNA kit for the unpooled samples where carrier was added to the TRIzol extraction procedure

There is not a method currently available that can evaluate the exact amount or quality of small RNA. Extracellular miRNAs are less than 1 % of the total RNA recovered [40], and since both total extracellular miRNA and RNA are recovered, the miRNA concentration is prone to overestimation if total RNA is degraded during the isolation process. Therefore, standard methods for measurement of the RNA yield and quality are not adequate for biofluid samples. The quantification of miRNAs in blood is further complicated by the high protein content and low RNA concentrations in plasma and serum. Even with the use of carrier molecules to improve isolation [49, 54], yields are often at or below the threshold for accurate quantification by spectrophotometric analyses [42]. Spectrophotometric measurement of RNA at OD 260 nm becomes impossible for some of these samples because of the presence of carrier RNA. Even without using carrier in the extraction, the concentration of the RNA obtained was still too low for spectrophotometric analysis since each of our extracts had an RNA concentration of less than 10 ng/µL (data not shown). Recently, plasma samples were shown to yield quantifiable RNA concentrations (only when a carrier was not added during the extraction procedure) when the plasma input was raised to 6 ml [55] using Qiagen miRNeasy Serum/Plasma Kit for extraction. When the miRNA yield and concentration are sufficient, quality assessment of the extraction method can be performed by capillary electrophoresis using the Agilent 2100 Bioanalyzer. The TRIzol extracts had ratios of miRNA to small RNA at less than 40 % from pooled as well as unpooled samples (Fig. 3a). Addition of carrier during TRIzol extraction increased the miRNA to small RNA ratio except when carrier yeast tRNA was used in extracting the pooled samples. ExoQuick Exosome precipitation prior to the TRIzol procedure did not yield greater amounts of miRNA as compared to the TRIzol procedure alone. The miRNeasy Mini Kit appeared to guarantee a constant amount of miRNA extracted across all corresponding samples.

The Agilent 2100 Bioanalyzer system quantifies RNA using an algorithm based on ribosomal RNA detection and assigns an RNA Integrity Number (RIN) to demonstrate RNA quality. However, this algorithm is not applicable to quantification of miRNA extracted from serum samples. Recently, it was reported that the percentage of RNA fragments >200 nt in a sample (termed the “DV 200” value) is a more reliable predictor of RNA quality when analyzing highly-degraded RNA sample quality as compared to the traditional integrity score or value [56].

There are conflicting opinions on whether biological carrier RNAs might be responsible for non-specific hybridization or amplification in downstream analytics [44]. Additionally, degradation of carrier RNA during RNA isolation could lead to inaccurate quantification for downstream applications. It is important to use highly purified carrier or co-precipitant. We did not observe any smearing in the extracts isolated using glycogen, whereas we detected a smear for the extracts where MS2 RNA and yeast tRNA were used as carrier molecules (Fig. 3b), reflecting carrier degradation during precipitation or background isolated with the extraction procedure. The examination of individual miRNAs by qRT-PCR is recommended but was not attempted for our extracts because of limited availability.

It is known that RNA yield is affected by the specific purification methods used [53, 57]. The choice of methodology for miRNA extraction from bodily fluids depends mainly on the available initial volume of samples and the methods for subsequent analyses, and most of the variables are study- and investigator-dependent. Our data shows small RNA has been preserved in the DoDSR archived samples and each protocol tested was successful in extracting miRNA.

### Small RNA cDNA library preparation

Studies have attempted to detect plasma/serum miRNA using microarrays and qRT-PCR [58]. Recently, there are reports that miRNAs in the circulatory system have been characterized using a deep-sequencing approach for serum and plasma [59, 60]. The success of small RNA sequencing depends most on the composition of the library being free of undesirable degradation remnants or naturally abundant short ribosomal RNA fragments [61]. Extractions of larger volumes of plasma have yielded 35 ng of total RNA in preparation for sequencing [55]. As per Illumina guidelines, it is recommended to use the maximum amount of total RNA (1 µg) in preparation for sequencing. Since we could not determine quantities of small RNA, we prepared our libraries with a fixed starting volume of extracted RNA. We did not see a discrete band for the miRNA library, but the library was size-selected for isolation and a High Sensitivity DNA Assay on the Agilent 2100 Bioanalyzer revealed that cDNA libraries of the correct size were generated with adaptor-ligated constructs, which signified the successful amplification of mature miRNAs. The resulting small RNA library was pooled and loaded on a flow cell in a random manner on the Illumina HiScan sequencing run.

## Sequencing results

### Read mapping/annotation and expression profiling-data processing

**Fig. 4:**
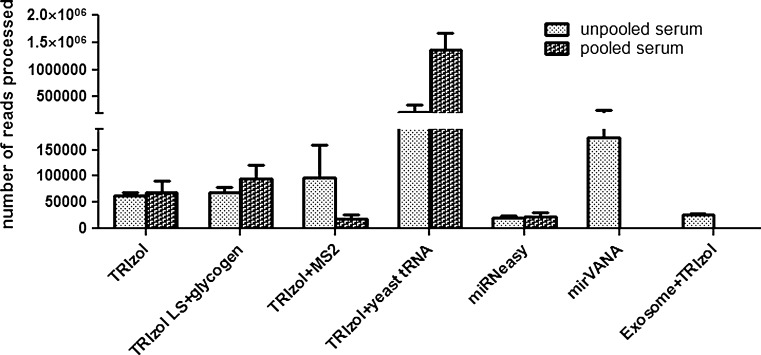
The total number of reads: The reads processed after quality assessment, filtering and adapter trimming for the unpooled as well as for the pooled samples. The *error bar* represents the *standard error* of mean

Our data analysis pipeline performs preprocessing of reads, aligns sequence reads against reference genome assembly, and quantifies miRNAs, followed by a quality control step. The alignment step generates alignment files and HTML files with mapping statistics for the percentage of reads mapped out of total number of reads against the reference genome/miRBase. Pooling has been used to examine the general characteristics of the reads [62] and for this reason, we used pooled samples in our study. We found similar numbers of processed reads from pooled as well as unpooled samples when TRIzol and miRNeasy methods were utilized. The number of processed reads using each procedure is shown in Fig. 4. TRIzol with glycogen had comparable reads from the pooled (94,203 reads) as well as unpooled (69,146 reads) samples. No consistency in processed reads was observed between pooled and unpooled samples when bacteriophage MS2 or yeast tRNA was added. Yeast tRNA had 6 times greater processed reads from pooled samples as compared to unpooled samples whereas bacteriophage MS2 had around 5 times fewer processed reads from pooled samples as compared to unpooled samples. This indicates that the addition of carrier RNA can lead to non-uniformity in general. We were not able to identify any study that has used carrier-supplemented RNA for sequencing. The consistency in similar amount of reads from glycogen supplemented samples might be attributed to using TRIzol LS during extraction and needs to be investigated. The TRIzol LS differs from standard TRIzol in concentration and is formulated to use with liquid samples such as serum. However, when quantifying digital gene expression, potential modification of the carrier nucleic acid may result in subsequent sequencing of carrier DNA or RNA [63]. Exosome precipitation prior to RNA extraction yielded a lower number of processed reads as compared to yields from extraction by the TRIzol procedure without a carrier. Furthermore, it has been stressed that the quantity of carrier used is crucial in RNA yield, as carrier concentration can mask the quantity of extracted RNA and affect the accuracy of the quantification and quality of the analysis [50].

### Size distribution of small RNA

**Fig. 5:**
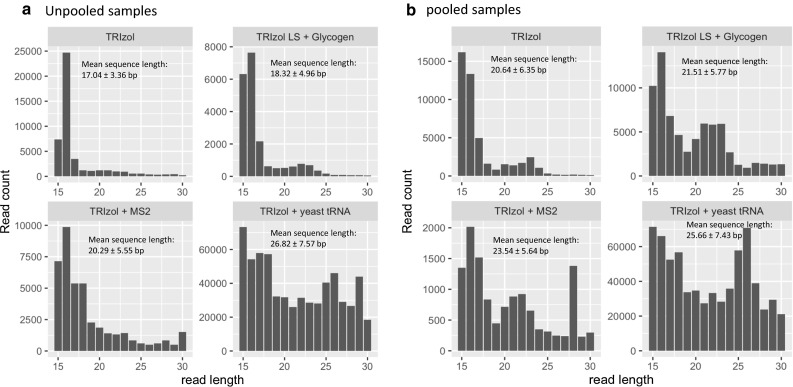
Read distribution plots, TRIzol with and without addition of carrier: Size distribution of total sRNA range from 15 to 37 nt. **a** Unpooled serum samples. **b** Pooled serum samples

The miRNA profile was compared between different extraction methods with and without, as well as before and after, the addition of carrier (results from TRIzol extractions shown in Fig. 5), and we observed a read length of 20–25 nt from pooled serum samples (Fig. 5b). The number of sequences is significantly higher, i.e. 69,804 in unpooled samples and 128,364 in pooled samples where carrier yeast tRNA was added during the isolation procedure. The TRIzol only procedure had 48,020 and 19,008 sequences from unpooled and pooled samples respectively. The TRIzol with glycogen procedure had only 5620 sequences from unpooled samples which was lowest among these different carriers. In case of pooled samples TRIzol with MS2 had 9176 sequences, the lowest among rest of the supplemented carriers. The addition of yeast tRNA also yielded much larger-sized fragments, followed by fragment sizes obtained using glycogen and bacteriophage MS2. However, from the unpooled samples (Fig. 5a), the histogram shows the peak around 15–20 nt read length, with and without carrier supplementation. The method with addition of yeast tRNA gave the maximum number of sequences. All other methods except the TRIzol supplemented with yeast tRNA had read lengths from 15 to 30 nt, but the TRIzol with yeast tRNA had a larger range of read length, 15–38 nt. Longer tRNA read lengths between 26 and 32 nt could be attributed to degraded fragments because of carrier introduction during extraction procedure or piwi-interacting RNA (piRNA), known to be longer in length than miRNA [55]. It has been reported that the normal range of miRNA is 18–24 nt [64], although most are 22–23 nt [65].

### Proportion of miRNAs

**Fig. 6:**
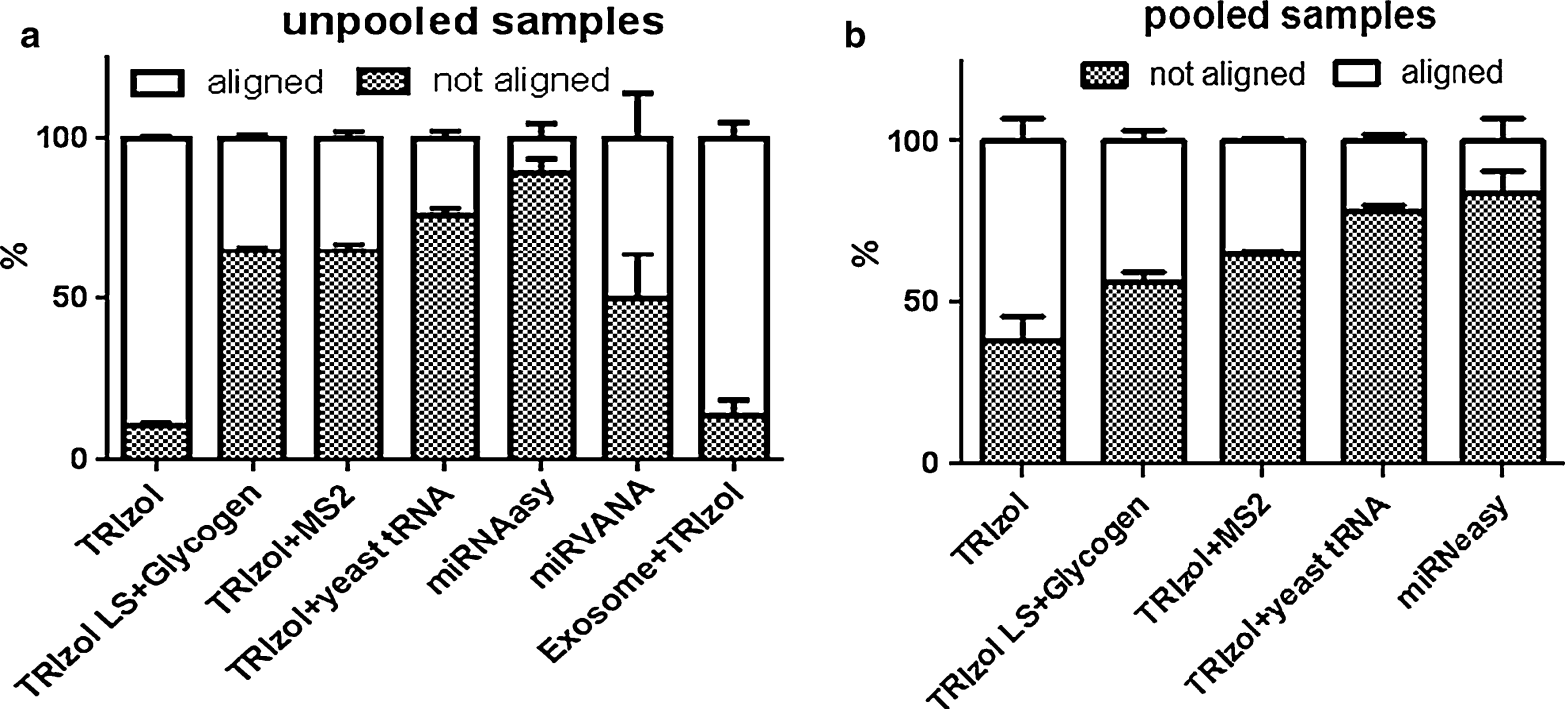
Annotated miRBase sequences: Processed reads were aligned to miRBase and reads aligned are represented for **a** Unpooled serum samples **b** Pooled serum samples

The count matrix of total read counts was generated for each annotated miRNA. Reads were mapped using an alignment mode that allows zero mismatches for the readlength of 22 nt for high-quality read ends, and stringent alignment options were selected in the aligner to obtain the best alignments which trimmed 10 bases from the low-quality (3′) end of each read before alignment. The good quality processed reads were aligned to mature miRBase and only a proportion of sequences mapped to the database, as shown in stacked bar plot (Fig. 6). The TRIzol extraction procedure gave the maximum percentage of aligned reads, 90 % from unpooled samples and ~62 % from pooled samples. The un-aligned read fraction is shown as the shaded area in the bar plot whereas white region is the fraction of aligned sequences. The miRNeasy extractions produced fewer than 17 % aligned reads from both of the sample types. The mirVANA extraction procedure resulted in 50 % of aligned sequences from unpooled samples. The TRIzol extraction with yeast tRNA as a carrier yielded fewer than 25 % aligned sequences from pooled as well as unpooled samples. TRIzol extraction with bacteriophage MS2 carrier gave 36 % aligned sequences from both of the sample types. Addition of glycogen during the extraction procedure also led to aligned sequences of fewer than 44 % from pooled and 35 % from unpooled samples. The percentage of reads mapped to miRBase shows 40–50 % from pooled samples and 80–90 % from unpooled samples. Most of the protocols, except miRNeasy, displayed 20–50 % higher percentage of reads mapping to miRBase from unpooled samples in comparison to reads from pooled samples. Although TRIzol with yeast tRNA and TRIzol with MS2 yielded the maximum numbers of processed reads, these extraction procedures produced the lowest percentage of aligned sequences to miRBase, revealing that degradation of the carrier RNA during extraction procedure contributed to the small RNA isolated. The miRNA count generated for the each sample is shown in Supplemental Table 1. Correlation analysis of miRNA counts within each extraction procedure was performed and replicates within the same extraction procedure had a very good correlation coefficient ranging from 0.76 to 0.99. For the pooled samples correlation coefficient between replicates was ranging from 0.76 to 0.94 whereas the replicates from unpooled samples had a correlation coefficient in a range from 0.88 to 0.99. The correlation analysis across methods was done on pooled samples and we found out that TRIzol with ytRNA had no correlation with TRIzol, TRI-zol + glycogen, TRIzol + MS2 and miRNeasy procedures (coefficient 0.01–0.05). The correlation coefficient for TRIzol with TRIzol + MS2 (0.39), TRIzol + glycogen (0.89) and miRNeasy (0.66), respectively.While these within and across method comparisons give some insights into their relative performances, without ground-truth knowledge of miRNA content in these degraded samples, we would further like to separate the signal from the noise in our future studies. This study is an exploratory phase for detection miRNA from the samples stored in compromised storage conditions and the data is promising, considering the signal that may have noise in the form of false positives. The reads that were not mapped to miRBase might be due to small RNAs in the samples that are not yet annotated and to filtering out reads of shorter lengths from maximum reads before mapping. Also, since RNAs are exposed to a high content of RNases in serum, RNA released from apoptotic cells, active release, or carriers could be degraded into pieces with an equivalent size range that would superimpose onto the length distribution profile.

### RNA species count

**Fig. 7:**
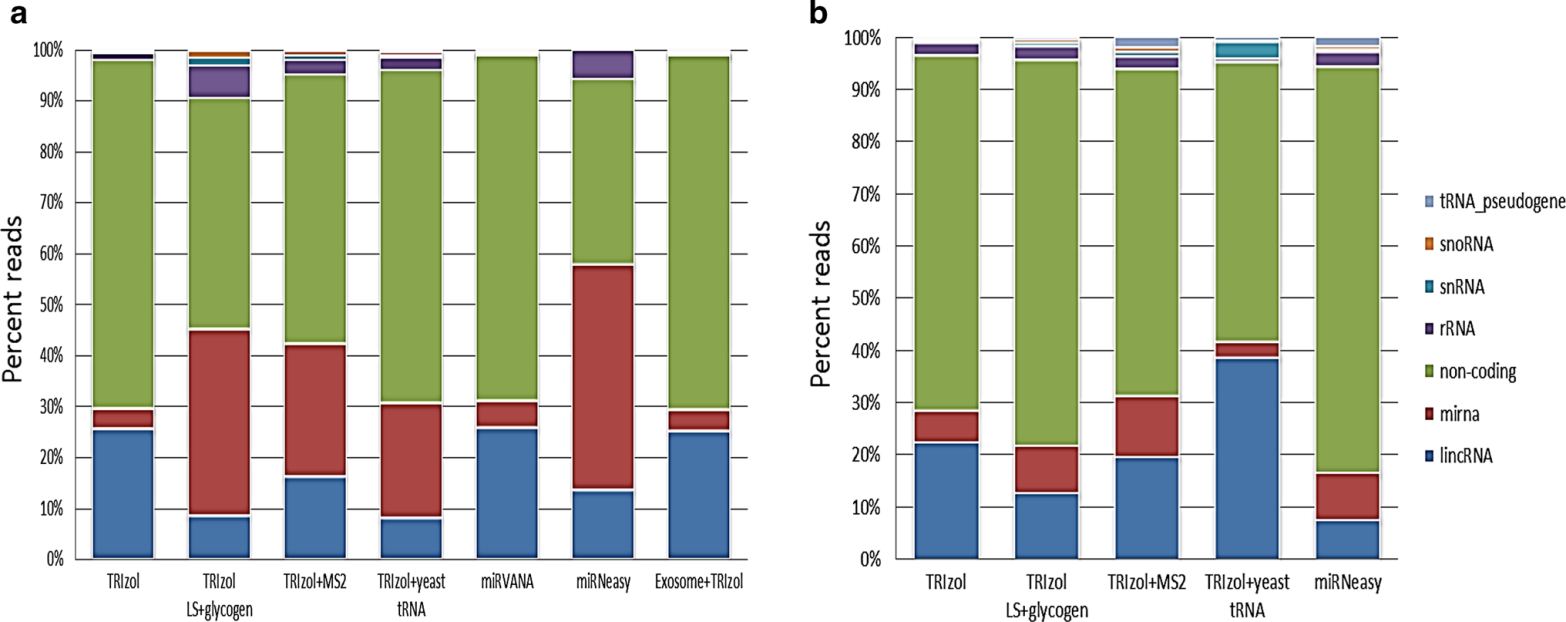
Reads aligned to other RNA species: The processed reads were aligned to the Ensembl database and the percent of reads aligning to different RNA species is shown. **a** Unpooled samples **b** Pooled samples. Chi square comparisons for small RNA species were significant at p value; considering the variability within methods, Kruskal–Wallis rank sum analysis was performed. Unpooled samples had Chi squared = 99.021, df = 11, *p*-value = 2.79e–16 and pooled samples had Chi squared = 191.56, df = 11,* p*-value < 2.2e–16. The p-value turns out to be nearly zero and thus rejecting null hypothesis and small RNA species population are not identical in different methods

More than 95 % of the reads from most of the unpooled sample types were annotated with coding sequences. The TRIzol with carrier yeast tRNA procedure had 81 % of the reads in coding regions whereas TRIzol with MS2 had 92 % aligned to coding regions (data not shown). From the pooled samples, 90 % of the reads were in the coding regions for all the procedures tested. We did not observe an increase in reads mapping to the tRNA as a result of the extraction procedure including yeast tRNA as a carrier. The coding RNA species were the largest proportion when mapped to the genome, and furthermore, other RNA species involved in many biological processes were identified among the isolated RNAs. The proportions of the RNA species other than coding RNA are shown in Fig. 7: long intergenic non-coding RNA (lincRNA), miRNA, ribosomal RNA (rRNA), followed by small nuclear (sn) RNA, small nucleolar (sno) RNA, tRNA pseudogenes, and other noncoding RNA. From unpooled samples, the proportion of lincRNA ranged from 8 to 26 % with the TRIzol, mirVANA and Exosome precipitation with TRIzol extraction procedures yielding the highest numbers. The proportion of miRNA obtained from unpooled samples increased with use of the TRIzol procedures where carrier was added (Fig. 7a). For the pooled samples, the carrier did not result in a dramatic increase in miRNA proportion (Fig. 7b). This demonstrates again that there are a number of variables associated at different steps, from sample collection through data analysis.

### Serum miRNA isolated in common by different methods

We analyzed the associations among different replicates extracted by different miRNA isolation methods, and all of the samples were positively correlated to each other for pooled as well as for unpooled samples. The correlation of pooled samples ranged from 76 to 94 %, whereas for unpooled samples, it ranged from 88 to 99 %. We found that TRIzol with yeast tRNA gave a very poor correlation for miRNA counts with the rest of the procedures, for pooled as well as unpooled samples (data not shown). It has been reported that the addition of carrier tRNA during RNA or DNA extraction can lead to false positives during hybridization-based assays due to carry over of contaminating nucleic acids [66]. We can identify miRNA isolated using the TRIzol-only method and the modified TRIzol methods that we assessed were sufficient for obtaining miRNA reads from the serum “test samples.” Weber et al., [36] quantified detectable miRNA from plasma using Qiagen qPCR assays and listed the top 20 most abundantly expressed miRNA in plasma. The miRNA counts observed from our data are presented in Supplemental Table 1.

**Fig. 8:**
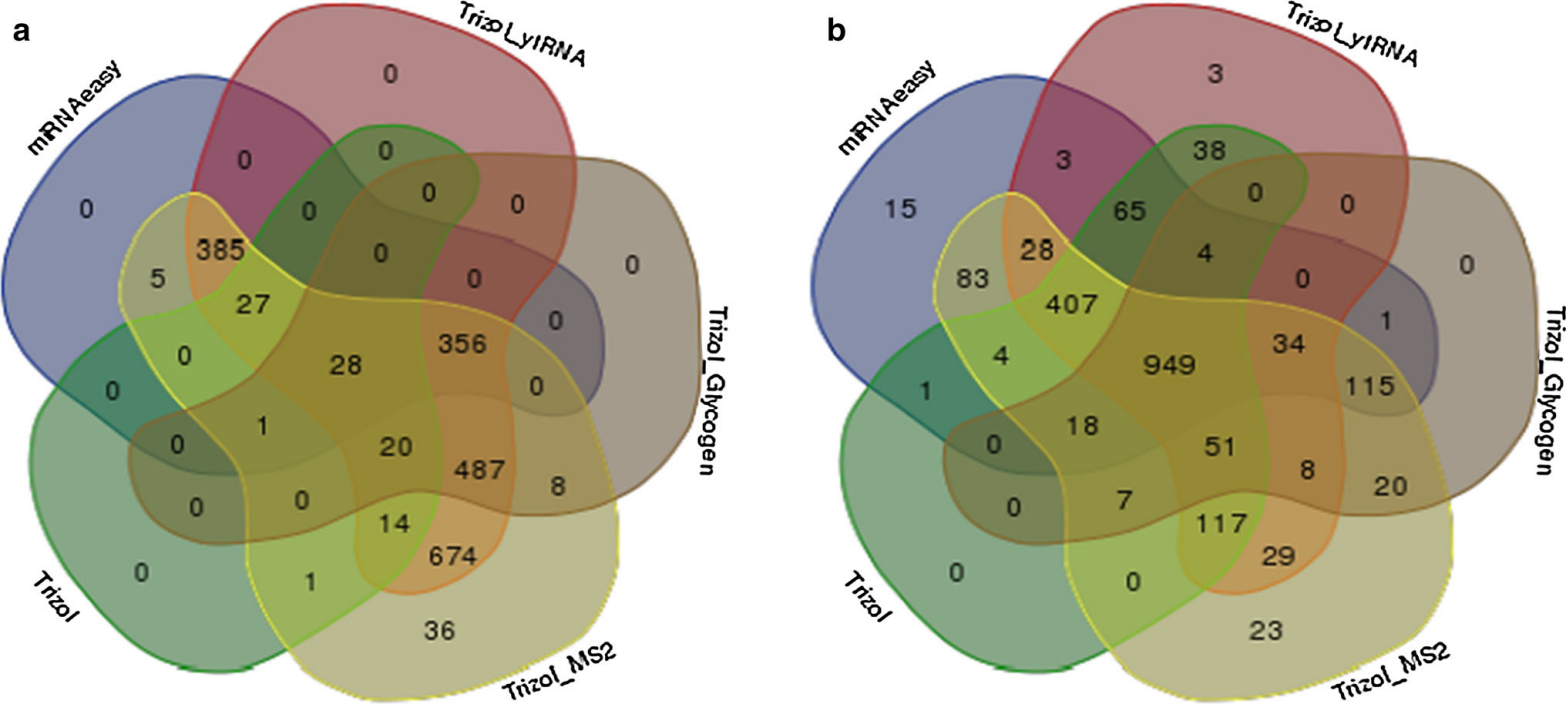
*Venn diagram* representing number of miRNA isolated in common: The miRNAs identified from the triplicate samples using the different isolation procedures have been appended into one list and compiled into a *Venn diagram* representing the common miRNAs between the different isolation procedures **a** Unpooled samples **b** Pooled samples

Since we were interested in discovering whether different extraction methods yield extracts that share miRNAs, the assembled list from three replicates was utilized to determine the common miRNAs among the extraction procedures (Fig. 8a, b). From the unpooled samples, all of the protocols shown in Fig. 8a produced 28 miRNAs in common, whereas TRIzol with glycogen, TRIzol with MS2, TRIzol with yeast tRNA, and miRNeasy protocols share 356 common isolated miRNAs. As the Venn diagram illustrates, the extraction with bacteriophage MS2 carrier yielded the maximum number of miRNAs overlapping with those from the rest of the procedures for both sets of samples. The next protocol that showed maximum overlap is miRNeasy, although the number of processed reads was very low for this method. There were no unique miRNAs detected from the TRIzol and TRIzol with glycogen methods from pooled as well as unpooled samples. However, TRIzol with yeast tRNA had three unique miRNAs among the pooled samples. From the pooled samples, all of the protocols yielded a total of 949 miRNAs in common with read counts ≥6.0 _log_2 counts per million (logCPM) values (Fig. 8b). Excluding the TRIzol with glycogen protocol, the TRIzol-only, TRIzol with MS2, TRIzol with yeast tRNA, and miRNeasy protocols still gave 407 miRNAs in common. Since the pooled “test samples” are derived from a large pool, we expect to see an increase in the number of common miRNAs obtained by all of the extraction procedures. We further analyzed the data to observe if there is any particular purification method that is affecting enrichment of certain miRNA families. The miRNA counts were TMM normalized and the expression values for all miRNA families for the procedures used for pooled samples is shown as Supplemental Table 2. The miRNeasy extraction method had largest numbers of miRNA’s (~40) that are not represented in samples when compared to other methods. Some of the miRNA that were not expressed in miRNeasy extraction method were miR-606, miR-1290, miR586, miR577, miR384, miR297, miR-5697, and miR-4328. Some of the miRNA families were consistent among different procedures, such as miR-370, miR4428, miR765, miR3168, miR5687, etc. with higher expression numbers for logCPM threshold cutoff greater than 6. The TRIzol with glycogen had lowest expression for many of the miRNA with expression values lower than 6 logCPM threshold cutoff. In unpooled samples, only the TRIzol extraction with addition of bacteriophage MS2 method yielded unique miRNAs [36].

## Conclusion

The study of miRNAs in serum from the DoDSR has the potential to reveal insights into diseases of military relevance. The circulating miRNAs are stable for long periods of time, but there remain many challenges for identifying these miRNAs, from the extraction procedures to NGS. We were able to extract total RNA in archived serum samples of limited availability using a variety of isolation methods available, thus enabling the sequencing of small RNA and analysis of circulating, cell-free miRNAs with good performance data. Our analyses showed that TRIzol with and without addition of glycogen or miRNeasy columns can be the methods of choice for isolating miRNAs from archived serum samples.

## Electronic supplementary material

Below is the link to the electronic supplementary material.

Supplementary material 1 (XLSX 1598 kb)

Supplementary material 2 (XLSX 81 kb)

## References

[1] Krol J, Loedige I, Filipowicz W (2010). The widespread regulation of microRNA biogenesis, function and decay. Nat Rev Genet.

[2] Valencia-Sanchez MA, Liu J, Hannon GJ, Parker R (2006). Control of translation and mRNA degradation by miRNAs and siRNAs. Genes Dev.

[3] Ameres SL, Zamore PD (2013). Diversifying microRNA sequence and function. Nat Rev Mol Cell Biol.

[4] Ha M, Kim VN (2014). Regulation of microRNA biogenesis. Nat Rev Mol Cell Biol.

[5] Cai Y, Yu X, Hu S, Yu J (2009). A brief review on the mechanisms of miRNA regulation. Genomics Proteomic Bioinform.

[6] Lewis BP, Shih IH, Jones-Rhoades MW, Bartel DP, Burge CB (2003). Prediction of mammalian microRNA targets. Cell.

[7] Bartel DP (2009). MicroRNAs: target recognition and regulatory functions. Cell.

[8] Cortez MA, Bueso-Ramos C, Ferdin J, Lopez-Berestein G, Sood AK, Calin GA (2011). MicroRNAs in body fluids–the mix of hormones and biomarkers. Nat Rev Clin Oncol.

[9] Vasilatou D, Papageorgiou S, Pappa V, Papageorgiou E, Dervenoulas J (2010). The role of microRNAs in normal and malignant hematopoiesis. Eur J Haematol.

[10] Zhou B, Wang S, Mayr C, Bartel DP, Lodish HF (2007). miR-150, a microRNA expressed in mature B and T cells, blocks early B cell development when expressed prematurely. Proc Natl Acad Sci USA.

[11] Reid G, Kirschner MB, van Zandwijk N (2011). Circulating microRNAs: association with disease and potential use as biomarkers. Crit Rev Oncol Hematol.

[12] MacLellan S, MacAulay C, Lam S, Garnis C (2014). Pre-profiling factors influencing serum microRNA levels. BMC Clinical Pathology.

[13] Keller A, Leidinger P, Bauer A, Elsharawy A, Haas J, Backes C, Wendschlag A, Giese N, Tjaden C, Ott K, Werner J, Hackert T, Ruprecht K, Huwer H, Huebers J, Jacobs G, Rosenstiel P, Dommisch H, Schaefer A, Muller-Quernheim J, Wullich B, Keck B, Graf N, Reichrath J, Vogel B, Nebel A, Jager SU, Staehler P, Amarantos I, Boisguerin V, Staehler C, Beier M, Scheffler M, Buchler MW, Wischhusen J, Haeusler SF, Dietl J, Hofmann S, Lenhof HP, Schreiber S, Katus HA, Rottbauer W, Meder B, Hoheisel JD, Franke A, Meese E (2011). Toward the blood-borne miRNome of human diseases. Nat Methods.

[14] Ichikawa M, Akiyama H (2013). A combination of extraction reagent and DNA microarray that allows for the detection of global miRNA profiles from serum/plasma. Methods Mol Biol.

[15] Chen X, Ba Y, Ma L, Cai X, Yin Y, Wang K, Guo J, Zhang Y, Chen J, Guo X, Li Q, Li X, Wang W, Zhang Y, Wang J, Jiang X, Xiang Y, Xu C, Zheng P, Zhang J, Li R, Zhang H, Shang X, Gong T, Ning G, Wang J, Zen K, Zhang J, Zhang C-Y (2008) Characterization of microRNAs in serum: a novel class of biomarkers for diagnosis of cancer and other diseases. Cell research 18 (10):997-1006. doi:http://www.nature.com/cr/journal/v18/n10/suppinfo/cr2008282s1.html 10.1038/cr.2008.282 18766170

[16] Grasedieck S, Scholer N, Bommer M, Niess JH, Tumani H, Rouhi A, Bloehdorn J, Liebisch P, Mertens D, Dohner H, Buske C, Langer C, Kuchenbauer F (2012). Impact of serum storage conditions on microRNA stability. Leukemia.

[17] Arroyo JD, Chevillet JR, Kroh EM, Ruf IK, Pritchard CC, Gibson DF, Mitchell PS, Bennett CF, Pogosova-Agadjanyan EL, Stirewalt DL, Tait JF, Tewari M (2011). Argonaute2 complexes carry a population of circulating microRNAs independent of vesicles in human plasma. Proc Natl Acad Sci USA.

[18] Zhu W, Qin W, Atasoy U, Sauter ER (2009). Circulating microRNAs in breast cancer and healthy subjects. BMC Res Notes.

[19] Keller A, Leidinger P, Gislefoss R, Haugen A, Langseth H, Staehler P, Lenhof HP, Meese E (2011). Stable serum miRNA profiles as potential tool for non-invasive lung cancer diagnosis. RNA Biol.

[20] Rounge TB, Lauritzen M, Langseth H, Enerly E, Lyle R, Gislefoss RE (2015). microRNA biomarker discovery and high-throughput DNA sequencing are possible using long-term archived serum samples. Cancer Epidemiol Biomark Prev.

[21] Thomson JM, Parker J, Perou CM, Hammond SM (2004). A custom microarray platform for analysis of microRNA gene expression. Nat Methods.

[22] Miska EA, Alvarez-Saavedra E, Townsend M, Yoshii A, Sestan N, Rakic P, Constantine-Paton M, Horvitz HR (2004). Microarray analysis of microRNA expression in the developing mammalian brain. Genome Biol.

[23] Mardis ER (2008). The impact of next-generation sequencing technology on genetics. Trends Genet.

[24] Morozova O, Marra MA (2008). Applications of next-generation sequencing technologies in functional genomics. Genomics.

[25] Zhang J, Gao Y, Yu M, Wu H, Ai Z, Wu Y, Liu H, Du J, Guo Z, Zhang Y (2015). Retinoic acid induces embryonic stem cell differentiation by altering both encoding RNA and microRNA expression. PLoS One.

[26] Rai G, Rai R, Saeidian A, Rai M (2015). Microarray to deep sequencing: transcriptome and miRNA profiling to elucidate molecular pathways in systemic lupus erythematosus. Immunol Res.

[27] Langmead B, Trapnell C, Pop M, Salzberg S (2009). Ultrafast and memory-efficient alignment of short DNA sequences to the human genome. Genome Biol.

[28] Flicek P, Amode MR, Barrell D, Beal K, Billis K, Brent S, Carvalho-Silva D, Clapham P, Coates G, Fitzgerald S, Gil L, Girón CG, Gordon L, Hourlier T, Hunt S, Johnson N, Juettemann T, Kähäri AK, Keenan S, Kulesha E, Martin FJ, Maurel T, McLaren WM, Murphy DN, Nag R, Overduin B, Pignatelli M, Pritchard B, Pritchard E, Riat HS, Ruffier M, Sheppard D, Taylor K, Thormann A, Trevanion SJ, Vullo A, Wilder SP, Wilson M, Zadissa A, Aken BL, Birney E, Cunningham F, Harrow J, Herrero J, Hubbard TJP, Kinsella R, Muffato M, Parker A, Spudich G, Yates A, Zerbino DR, Searle SMJ (2014). Ensembl 2014. Nucleic Acids Res.

[29] Kozomara A, Griffiths-Jones S (2014). miRBase: annotating high confidence microRNAs using deep sequencing data. Nucleic Acids Res.

[30] Kozomara A, GriffithsJones S (2011). miRBase: integrating microRNA annotation and deep-sequencing data. Nucleic Acids Res.

[31] Griffiths-Jones S, Saini HK, van Dongen S, Enright AJ (2008). miRBase: tools for microRNA genomics. Nucleic Acids Res.

[32] Griffiths-Jones S, Grocock RJ, van Dongen S, Bateman A, Enright AJ (2006). miRBase: microRNA sequences, targets and gene nomenclature. Nucleic Acids Res.

[33] Li H, Handsaker B, Wysoker A, Fennell T, Ruan J, Homer N, Marth G, Abecasis G, Durbin R (2009). The sequence alignment/map format and SAMtools. Bioinformatics.

[34] Quinlan AR, Hall IM (2010). BEDTools: a flexible suite of utilities for comparing genomic features. Bioinformatics.

[35] Schmieder R, Edwards R (2011). Quality control and preprocessing of metagenomic datasets. Bioinformatics.

[36] Weber JA, Baxter DH, Zhang S, Huang DY, Huang KH, Lee MJ, Galas DJ, Wang K (2010). The microRNA spectrum in 12 body fluids. Clin Chem.

[37] Wang K, Yuan Y, Cho J-H, McClarty S, Baxter D, Galas DJ (2012). Comparing the MicroRNA spectrum between serum and plasma. PLoS One.

[38] Kim DJ, Linnstaedt S, Palma J, Park JC, Ntrivalas E, Kwak-Kim JY, Gilman-Sachs A, Beaman K, Hastings ML, Martin JN, Duelli DM (2012). Plasma components affect accuracy of circulating cancer-related microRNA quantitation. J Mol Diagn.

[39] Page K, Guttery DS, Zahra N, Primrose L, Elshaw SR, Pringle JH, Blighe K, Marchese SD, Hills A, Woodley L, Stebbing J, Coombes RC, Shaw JA (2013). Influence of plasma processing on recovery and analysis of circulating nucleic acids. PLoS One.

[40] Moldovan L, Batte KE, Trgovcich J, Wisler J, Marsh CB, Piper M (2014). Methodological challenges in utilizing miRNAs as circulating biomarkers. J Cell Mol Med.

[41] Kim YK, Yeo J, Kim B, Ha M, Kim VN (2012). Short structured RNAs with low GC content are selectively lost during extraction from a small number of cells. Mol Cell.

[42] Kroh EM, Parkin RK, Mitchell PS, Tewari M (2010). Analysis of circulating microRNA biomarkers in plasma and serum using quantitative reverse transcription-PCR (qRT-PCR). Methods.

[43] Li Y, Kowdley KV (2012). Method for microrna isolation from clinical serum samples. Anal Biochem.

[44] McAlexander MA, Phillips MJ, Witwer KW (2013) Comparison of methods for miRNA extraction from plasma and quantitative recovery of RNA from plasma and cerebrospinal fluid. Frontiers in Genetics 4. doi:10.3389/fgene.2013.00083 10.3389/fgene.2013.00083 PMC3655275 23720669

[45] Rekker K, Saare M, Roost AM, Kubo A-L, Zarovni N, Chiesi A, Salumets A, Peters M (2014). Comparison of serum exosome isolation methods for microRNA profiling. Clin Biochem.

[46] Khoury S, Ajuyah P, Tran N (2014). Isolation of small noncoding RNAs from human serum. J Vis Exp.

[47] Taylor CJ, Satoor SN, Ranjan AK, Pereira e Cotta MV, Joglekar MV (2012). A protocol for measurement of noncoding RNA in human serum. Exp Diabetes Res.

[48] Duy J, Koehler JW, Honko AN, Minogue TD (2015). Optimized microRNA purification from TRIzol-treated plasma. BMC Genom.

[49] Andreasen D, Fog JU, Biggs W, Salomon J, Dahslveen IK, Baker A, Mouritzen P (2010). Improved microRNA quantification in total RNA from clinical samples. Methods.

[50] Moret I, Sánchez-Izquierdo D, Iborra M, Tortosa L, Navarro-Puche A, Nos P, Cervera J, Beltrán B (2013). Assessing an improved protocol for plasma microRNA extraction. PLoS One.

[51] Wang QT, Xiao W, Mindrinos M, Davis RW (2002). Yeast tRNA as carrier in the isolation of microscale RNA for global amplification and expression profiling. Biotechniques.

[52] Remakova M, Skoda M, Faustova M, Vencovsky J, Novota P (2013). Validation of RNA extraction procedures focused on micro RNA expression analysis. Folia Biol (Praha).

[53] Masotti A, Caputo V, Da Sacco L, Pizzuti A, Dallapiccola B, Bottazzo GF (2009). Quantification of small non-coding RNAs allows an accurate comparison of miRNA expression profiles. J Biomed Biotechnol.

[54] Pegtel DM, Cosmopoulos K, Thorley-Lawson DA, van Eijndhoven MAJ, Hopmans ES, Lindenberg JL, de Gruijl TD, Würdinger T, Middeldorp JM (2010). Functional delivery of viral miRNAs via exosomes. Proc Natl Acad Sci.

[55] Spornraft M, Kirchner B, Haase B, Benes V, Pfaffl MW, Riedmaier I (2014). Optimization of extraction of circulating RNAs from plasma—enabling small RNA sequencing. PLoS One.

[56] Illumina (2014) Evaluating RNA Quality from FFPE Samples Pub. No. 470 2014-001

[57] Ach RA, Wang H, Curry B (2008). Measuring microRNAs: comparisons of microarray and quantitative PCR measurements, and of different total RNA prep methods. BMC Biotechnol.

[58] Chim SSC, Shing TKF, Hung ECW, T-y Leung, T-k Lau, Chiu RWK, Dennis Lo YM (2008). Detection and characterization of placental MicroRNAs in maternal plasma. Clin Chem.

[59] Wu Q, Lu Z, Li H, Lu J, Guo L, Ge Q (2011) Next-generation sequencing of MicroRNAs for Breast Cancer Detection. J Biomed Biotechnol. doi:10.1155/2011/597145 10.1155/2011/597145 PMC3118289 21716661

[60] Williams Z, Ben-Dov IZ, Elias R, Mihailovic A, Brown M, Rosenwaks Z, Tuschl T (2013). Comprehensive profiling of circulating microRNA via small RNA sequencing of cDNA libraries reveals biomarker potential and limitations. Proc Natl Acad Sci.

[61] Matts JA, Sytnikova Y, G-w Chirn, Igloi GL, Lau NC (2014). Small RNA library construction from minute biological samples. Methods Mol Biol.

[62] Dhahbi JM, Spindler SR, Atamna H, Boffelli D, Martin DIK (2014). Deep sequencing of serum small RNAs identifies patterns of 5′ tRNA half and YRNA fragment expression associated with Breast Cancer. Biomark Cancer.

[63] Thompson JF, Raz T, Milos PM (2011). Tag-based Next Generation Sequencing.

[64] Juan L, Tong H-l, Zhang P, Guo G, Wang Z, Wen X, Dong Z, Tian Y-p (2014) Identification and characterization of novel serum microRNA candidates from deep sequencing in cervical cancer patients. Sci Rep 4. doi:10.1038/srep06277http://www.nature.com/srep/2014/140903/srep06277/abs/srep06277.html#supplementary-information 10.1038/srep06277 PMC4152751 25182173

[65] Pritchard CC, Cheng HH, Tewari M (2012) MicroRNA profiling: approaches and considerations. Nat Rev Genet 13 (5):358-369. doi:http://www.nature.com/nrg/journal/v13/n5/suppinfo/nrg3198_S1.html 10.1038/nrg3198 PMC4517822 22510765

[66] Hengen PN (1996). Carriers for precipitating nucleic acids. Trends Biochem Sci.

